# Soil carbon storage under different types of arid land use in Algeria

**DOI:** 10.1007/s10653-024-02036-w

**Published:** 2024-07-17

**Authors:** Abderraouf Benslama, Fouzi Benbrahim, Lydia Rym-Gadoum, Ignacio Gómez-Lucas, Manuel Miguel Mordan-Vidal, Jose Navarro-Pedreño, Jaume Bech-Borrás

**Affiliations:** 1 Laboratory of Valuation and Conservation of Arid Ecosystems (LVCEA), Department of Biology, Faculty of Sciences Natural and Life, Earth and Universe Sciences, University of Ghardaïa, BP455, 47000 Bounoura, Ghardaïa Algeria; 2 Department of Agrochemistry and Environment, University Miguel Hernández of Elche, 03202 Elche, Spain; 3 Higher Normal School of Ouargla, Hai Ennasr, BP 398, 30000 Ouargla, Algeria; 4 Laboratory of Soil Sciences, Faculty of Biology, Plant Biology, University of Barcelona, Barcelona, Spain

**Keywords:** Arid conditions, Forestry, Loss on ignition method, Pastoral, Soil organic matter, Walkley–Black method

## Abstract

This study aims to assess the amount of organic carbon stored in soils, as it is an intention of knowing the sustainable soil management, by using two common methods for determining soil organic matter (SOM), namely oxidation with acidified wet dichromate (Walkley–Black method-WB) and loss on ignition (LOI). The study was carried with soil samples collected from a depth of 0 to 30 cm in the Saharan arid region of Ghardaïa (Algeria), with different land uses: agricultural, forest and pastoral. The results obtained from the LOI and WB methods were subjected to statistical analysis, and the relations between both methods were tested to investigate their relationship. The mean percentage of SOM values were 1.86, 2.42, 1.54 by using LOI, but, lower values of 0.34, 0.33, 0.36 were determined by using WB method, for agricultural, forest and pastoral soils respectively. A weak linear relationship between the two analytical procedures was obtained (R^2^ of 0.19 and 0.13 for agricultural and forest soils), while a medium relationship (R^2^ = 0.65) was found for pastoral soils when using linear adjustment. However, the opposite behaviour was found when we use the logarithmic adjustment. The study outcomes indicated discrepancies in the measurements of SOM values between the two methods, been higher those estimated with LOI. Finally, in order to identify the best methodology to measure soil organic matter in arid soils, more research is required in these extreme arid regions as they are a gap in world soil organic matter maps.

## Introduction

Soil as an essential carbon reservoir, plays a crucial factor mitigating the increase in concentration of Greenhouse Gases (GHGs) in the atmosphere, mainly by storing Soil Organic Carbon (SOC) in the soil profile. This is why their use as GHGs sink is at the heart of many sustainable development issues and objectives (Abera et al., 2021) and the most of these objectives are associated to land management under agriculture, forestry and pastoralism. Monitoring patterns in soil carbon storage and identifying controlling factors on both regional and global scales are crucial for predicting and mitigating the impact of soil carbon on global environmental change (Zhang et al., 2023). SOC is a vital element for the proper functioning and stability of the soils, exerting influence over their physical, chemical and biological properties (Bongiorno et al., 2019; Gualberto et al., 2023; Kooch et al., 2020). Enhancing Soil Organic Carbon (SOC), thus Soil Organic Matter (SOM) stocks, has been proposed as a viable strategy to mitigate climate change, with the additional advantage of improving soil properties, mainly the soil structure (Lal, 2016).

SOC can be assessed by measuring the Total amount of Organic Carbon (TOC) present in the soil, regardless of its origin or decomposition, although not all the soil organic carbon determined has the same ecological functions and the ability to be mineralized (Navarro-Pedreño et al., 2021). It is a key contributor to the carbon cycle and global warming mitigation, and varies spatially according to the soil and environmental properties. For ecosystem productivity and soil functions, soil carbon sequestration and soil organic matter stock are crucial (Thomaz and Kurasz, 2023). In certain ecosystems, this carbon can persist in the soil for an extended period in recalcitrant forms, resistant to degradation. However, the type and quality of carbon that can be stored in soils depend on local ecosystems and environmental conditions, land use and land cover (Boubehziz et al., 2020; Malik et al., 2023).

Land degradation poses a threat to the sustainability of human societies (Masoudi et al., 2021) and the effects of land use and land cover (LULC) changes have emerged as a critical concern for the scientifics addressing global climate change (Lambin & Geist, 2008). Several studies report positive or non-significant changes in SOC content in no-till crops, forests and pastures (Battaglia et al., 2022; Bogale et al., 2023). However, in the last five decades, ecosystems have undergone unprecedented and widespread modifications, surpassing any comparable period in history. This phenomenon is particularly notable in the Mediterranean region (Serra et al., 2008; Steffen et al., 2011), under arid and semi-arid conditions, where the soils has been cultivated for millennia like in the Fertile Crescent. Nowadays, these regions are close and surrounded by desert areas with low organic matter and salinization processes in soils. The effects are noticed and human-induced climate change has involved to soil loss and degradation, resulting in a global decline in soil carbon storage (Eaton et al., 2008), this impact has been particularly pronounced in recent decades in Mediterranean regions (Cerdá et al., 2010; Jerez et al., 2018). To cope with rising carbon dioxide (CO_2_) levels in the atmosphere, and to enhance the potential of soils to store carbon, systematic and scientific attention needs to be paid to land use processes (Dawson & Smith, 2007; Lal, 2004). At the same time, special attention is given for the methods used to determine SOC, SOM and TOC. The Loss on Ignition (LOI) method is a widely used technique for estimating the organic matter content of soils by measuring the mass loss of soil samples upon ignition. This method, stands as one of the most commonly employed techniques for determining the organic matter content present in soils (Hoogsteen et al., 2015). Additionally, the WB method is another conventional approach used to measure SOC in soils (Roper et al., 2019). Hence, we therefore used both methods to thoroughly assess the SOM and SOC content of soil samples. The sensitivity and accuracy obtained in the determinations of organic carbon content and, consequently, soil organic matter, can be a problem. But so are the differences in the estimates obtained by various methods, which lead to very different results and make comparison difficult.

SOM pertains to the organic component of soil that excludes plant and animal residues that have not undergone decomposition. However, most analytical techniques do not distinguish between residues that have been degraded and those that have not, as SOM is a dynamic and heterogeneous substance with a range in turnover time, carbon concentration, and particle size (USDA, 2009). That is why the soil environment and the climatic conditions have a major role to influence the type of organic matter stored. It must be understood that stabilized and recalcitrant organic matter in the soil is the one that has the greatest contribution to mitigating the negative effects of climate change because it remains stored in the soil, retaining carbon that could otherwise be released into the atmosphere.

In recent years, changes in land use, particularly agricultural intensification and deforestation, have significantly contributed to the global warming process by releasing CO_2_ emissions (Houghton & Hackler, 1999; Lambin et al., 2001; Ostle et al., 2009; Schulp et al., 2008). Land use change is considered the second major cause of CO_2_ emissions subsequent to fuel consumption (Waston and Verardo, 2000; IPCC, 2007). According to Bai et al. (2008), the main human pressures or factors affecting soil degradation include: (a) agricultural demands, (b) nutrient extraction, (c) waste disposal, (d) population growth, (e) intensive cultivation, (f) overgrazing and (g) excessive irrigation. This degradation affects soil in several ways and, one of the most important is the loss of organic carbon from the soil. Notwithstanding, the position of organic matter in the soil profile determines its storage possibilities and the interactions with an aerobic environment close to the atmosphere (topsoil), or in anaerobic environment at depth, reducing gases exchange with the atmosphere. This position in the soil profile has a great influence on the physical, chemical and biochemical reactions of the organic matter compounds that lead to and increase or decrease of SOC storage. The soil carbon at 0–0.2 m depth, is the main reservoir of soil organic carbon but it is affected by human activities, mainly by agriculture, i.e., tillage. The organic matter in topsoil is labile, with a tendency to decomposition and transformation. Nevertheless, the top 0.2 m of soil contains more than half of the carbon within the first meter of soil profile, and it is the site of 80 percent (%) of the flux (Metay et al., 2017), the interchange between the atmosphere and the soil. Deep SOC is frequently overlooked in management strategies or carbon inventories, particularly in North Africa (Bounouara et al., 2017). In general, organic matter located deeper in the soil profile tends to be more stable, it does not decompose easily, and has less interaction within the atmosphere.

In the case of Algeria, there are regions considered vulnerable in terms of soil degradation due to the organic carbon loss. However, it's crucial to acknowledge that there are few published works and data on these specific regions of Algeria (Boubehziz et al., 2020) especially in extreme arid zones, similarly this knowledge gap exists in other arid regions globally, posing challenges to comprehending the mechanisms that govern soil organic matter dynamics at global, regional and local scales. This indicates that research is required to determine the organic carbon content and comprehend the mechanisms affecting its sequestration, deposition on the soil, transformation and storage in these areas. Studies on SOM quantity, quality, and dynamics in Algeria are limited, often confined to a preliminary characterization (Dellal & Halitim, 1992). However, the first step to understand SOM dynamic in arid soils is to have a good method to measure/estimate the organic matter content in the soil profile. Our study aims to estimate and determine soil organic carbon stored under different types of land use in regions subject to an arid climate in a Saharan part of Algeria (region of Ghardaïa), offering a critical point of view regarding the methodology used to estimate soil organic matter and soil organic carbon.

## Materials and methods

### Study area

**Fig. 1:**
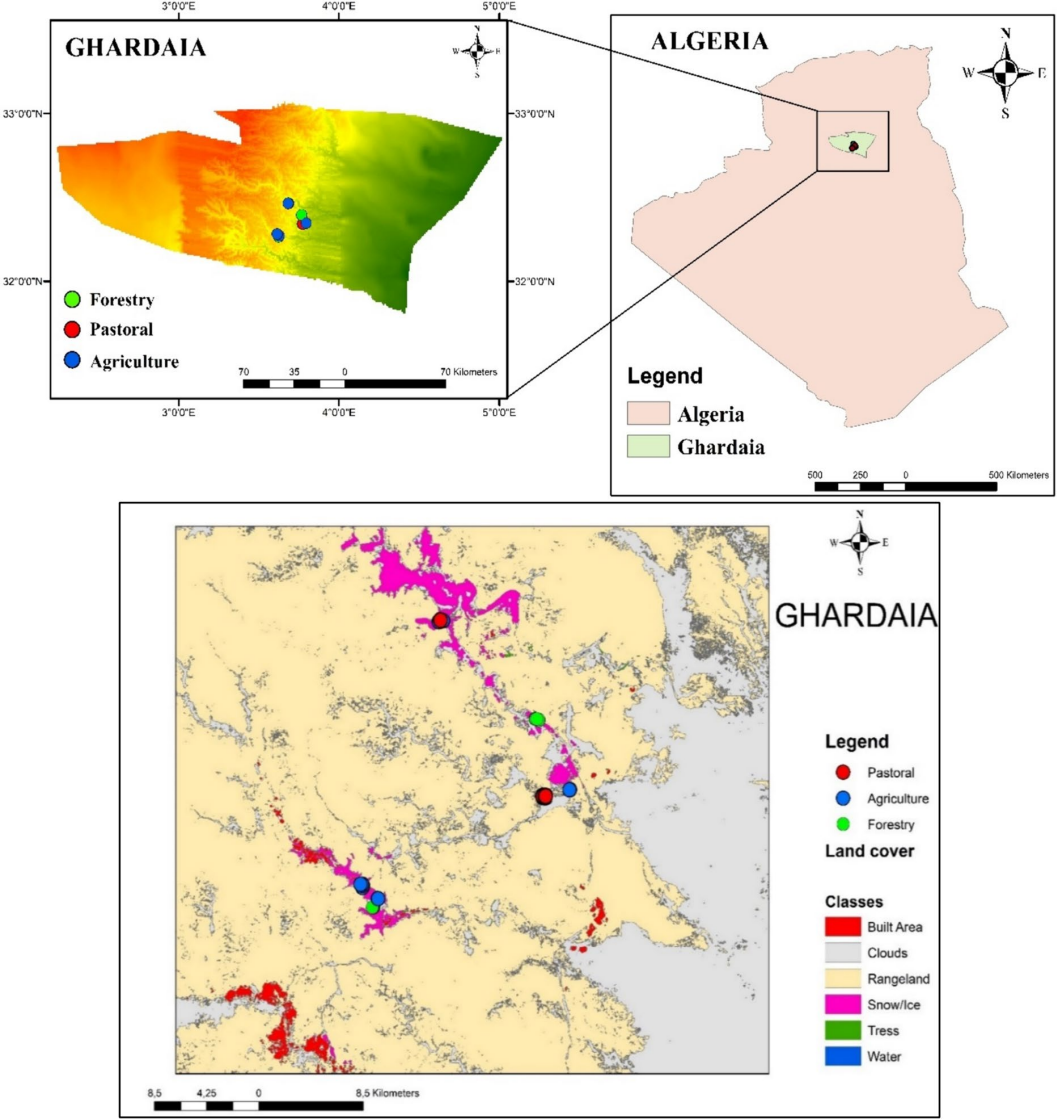
Geographic location of the study area in the region of Ghardaïa, Algeria

Our study, done during the first semester of 2023, was conducted in the province of Ghardaïa, located in central Algeria, between latitudes 29°19′ N and 32°57′ N and between longitudes 02°03′ and 04°54′ East. 29°19′ N–32°57′ N, 02°03′ E–04°54′. The area is presented (Fig. 1).

### Climate

The region's climate is characterized by a typically arid Saharan climate, with two seasons: a hot and dry season (April–September), and a temperate one (October–March), and a considerable difference between daily temperatures, with extreme temperature amplitudes between day and night reaching 15–16 degrees. The coldest month in this region is January, featuring a mean minimum temperature of 6.2 °C, while the hottest month is July, with a mean maximum temperature of 41.8 °C. Precipitation is limited and irregular, with values between 100 and 200 mm/year and episodes of torrential rain, and the evaporation is over 2000 mm per year (ONM, 2017).

### Soil sampling

**Fig. 2:**
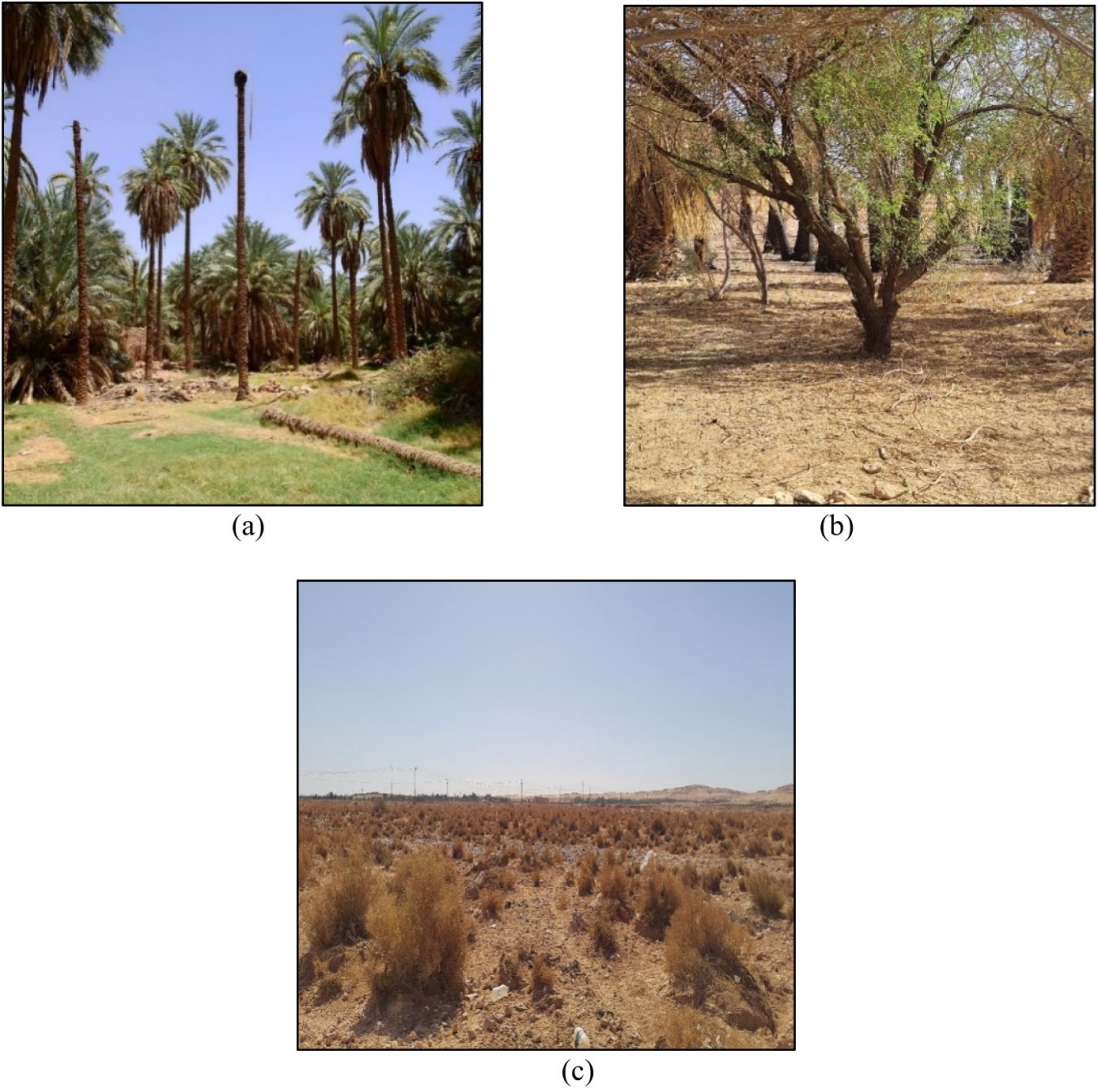
Sampling arid areas: agricultural soils (**a**), forest (**b**) and extensive pastoral soils (**c**)

All of the soil samples collected in the arid region of Ghardaïa, were classified as Arenosols in the World Reference Base for Soil Resources (IUSS, 2015). The samples were collected and located at 43 points distributed over three types of land use. 15 samples corresponding with agricultural soils supporting various crops, including date palm (Phoenix dactylifera), pomegranate (Punica granatum), and vegetable crops such as bottle gourd (Lagenaria siceraria). 13 samples were collected from forest land, featuring vegetation such as acacia and tamarisk (Tamaricaceae). Additionally, in the pastoral lands dedicated to dairy cows, 15 samples were collected from extensive soils dominated by esparto grass, including species like drinn (Aristida pungens) and cram-cram (Cenchrus biflorus) (Fig. 2).

These samples were collected for triplicate from a depth of 0–30 cm (top-soil and starting depth soil), transported to the laboratory, air-dried at room temperature, crushed and sieved to 2 mm. After the preparation of soil samples, soil texture was evaluated in accordance with USDA standards utilizing the Bouyoucos densimeter method (Bouyoucos, 1962), pH and EC were measured using United States Salinity Laboratory method (USSL Staff, 1954), and the equivalent calcium carbonate content (CaCO_3_) was assessed through the acid digestion method utilizing a calcimeter (Perry & Adams, 1978). Two different methods were used to determine soil organic matter. The methods used to estimate and determine soil organic matter were: loss on ignition (LOI) and the Walkley–Black method (WB) because both are highly recommended and, especially loss-on-ignition, which is an easy method that can be applied all over the world, facilitating the determination of soil organic matter. Replications of soils sampled at each point as well as the analytical measurements were done (three replications).

### Loss on ignition method

LOI is a commonly used technique for direct estimation of total organic matter (Bojko and Kabała, 2014; Mikutta et al., 2005; Raya-Moreno et al., 2017). It is usually based on burning the sample at high temperature (500–550 °C). However, to avoid gypsum and carbonate decomposition, a moderate temperature is recommended (Poot et al., 2009). The difference between the initial mass and the residual mass is used to estimate the amount of organic matter presented in the sample. This method has several advantages over other methods for organic carbon measurement, not least its simplicity and low cost. In our case, dry samples were placed overnight (8 h) in a muffle furnace at 375°C and the weight loss was calculated, without destroying inorganic carbon due to the moderate temperature used (Salehi et al., 2011). Organic Matter (OM) is calculated by using Eq. 1 (Salehi et al., 2011).

1 $$Soil\; organic\; matter\;{ }\left( {\% } \right)\; = \;\frac{{\left( {\left( {{\text{P}}1 - {\text{P}}0} \right) - \left( {{\text{P}}2 - {\text{P}}0} \right)} \right)}\times 100}{{\left( {{\text{P}}1 - {\text{P}}0} \right)}} $$

where;

P_0_: weight of empty crucible;

P_1_: final weight;

P_2_: weight of crucible containing ash.

### The Walkley–Black method

The Walkley–Black method determines the oxidable organic carbon with a sulfochromic attack, and after that, the value can be transformed into soil organic matter (Walkley & Black, 1934). The titration method is the procedure for determining soil organic matter after the acid attack produced by using potassium dichromate (K_2_Cr_2_O_7_) and concentrated sulfuric acid (H_2_SO_4_). The method used 0.5 g air-dried soil that was disposed in a 500 mL Erlenmeyer flask, adding 10 mL of K_2_Cr_2_O_7_ 1N, then, carefully, quickly add 20 mL of H_2_SO_4_, leave the flask to stand for 30 min (the exothermic reaction reaches temperatures around 120 °C). To stop the reaction, 200 mL water is added to the flask and 10 mL 85% phosphoric acid (H_3_PO_4_). The titration was done by using three to four drops of o-phenanthroline as indicator and Mohr's salt 0.5 M [(NH_4_)_2_Fe(SO_4_)_2_·6H_2_O] as titrating solution (FAO, 2019). Organic matter was calculated by using Eq. 2.

2 $$Soil \;Organic \;Carbon \% = \frac{{\left( {{\text{V}}\;{\text{ blank}} - {\text{V}}\;{\text{ sample}}} \right) \times {\text{MFe}}2{ } \times 0.003 \times 100 \times {\text{f*mcf}}}}{{\text{W}}}$$

where;

*V blank* = volume of titrant in blank, mL.

*Vsample* = volume of titrant in sample, mL.

*MFe*_2_ = (NH_4_)_2_Fe(SO_4_)_2_·6H_2_O solution, molarity.

0.003 = oxidized carbon (12 g C/mole × 1 mol K_2_Cr_2_O_7_/6 mol FeSO4 × 3 mol C/2 mol K_2_Cr_2_O_7_ × 1 L/1000 mL)

F = correction factor, 1. 3

W = weight of soil, g

mcf = moisture correction factor.

The SOC is usually multiplied by 1.724, “Van Bemmelen factor” which assumes that soil organic matter contains 58% of C (Pribyl, 2010; Shamrikova et al., 2022), to obtain the percentage of oxidizable organic matter, using Eq. 3 (Shamrikova et al., 2022).

3 $$\text{Oxidizable}\;\text{ soil}\;\text{ organic}\;\text{ matter}\;\%\; = \;{\text{soil}}\;{\text{ organic }}\;{\text{carbon }} \times 1.724$$

### Statistical analysis

Statistical analysis was carried out by using Excel Stat ©, the descriptive statistics for soil samples includes, maximum (max), minimum (min), mean, the coefficient of variation (CV) and standard deviation (St.dev.). The Kolmogorov–Smirnov test was used to test the normal distribution of the data.

To assess the degree of linear correlation between the two analytical methodologies to estimate organic matter (OM) Pearson’s correlation coefficient (R) was determined. The relationships between WB-OM and LOI-OM were studied applying simple linear regression as it is expected a direct relation between both procedures and the content of soil organic matter. However, the logarithmic adjustment was tested, as the disposition of the points suggested the possibility of a better adjustment by using this regression.

## Results and discussion

The mean values for soil samples taken from the three types of land use (agriculture, forestry, pastoralism) in the Ghardaïa region indicates soil texture are sandy-silty with pH varied 7.9–8.2, electrical conductivity varied 2.1–6.1 (dS/m) and carbonate varied 16.3–20.7 (%) Table 1.

**Table 1:** Mean value of the physico-chemical characteristics of soil

	Texture	pH	EC (dS/m)	Eq. CaCO_3_ (%)
Agricultural soils	Sandy-silty	8.2	5.2	16.3
Forest land	Sandy-silty	8.1	6.1	18.6
Pastoral lands	Sandy-silty	7.9	2.1	20.7

The descriptive statistics indicates for soil organic matter in the samples, by using the WB and LOI methods, are presented in Table 2, and that important differences were found in the results obtained considering both methodologies. The main difference was that SOM values were always over two to three times higher when using LOI to determine SOM than WB. This difference is too high to consider it only dependent of an overestimation with LOI or because of the use of the Van Bemmelen factor for the calculation of SOM from SOC, in the case of WB. As it is shown in Table 2, the mean percentage of SOM values, expressed in dry weight basis (% d.w.), were for the LOI 1.86, 2.42, 1.54, and for WB 0.34, 0.33 and 0.36, considering the three types of land use, agricultural, forest and pastoral soils respectively.

**Table 2:** Mean values and descriptive statistics of soil organic matter (% d.w.)

	Parameter	Agricultural	Forest	Pastoral
SOM WB	Mean	0.34	0.33	0.36
	Max	0.72	0.61	1.08
	Min	0.03	0.04	0.05
	St.dev	0.17	0.17	0.26
	CV(%)	0.50	0.51	0.72
SOM LOI	Mean	1.86	2.42	1.54
	Max	5.16	4.95	4.11
	Min	0.35	1.06	0.33
	St.dev	1.47	1.14	0.93
	CV(%)	0.79	0.47	0.60

*Max* maximum *Mi*n: minimum, *St.dev* standard deviation, *CV*% coefficient of variation

Low percentages of soil organic matter were obtained from the measurements of SOC by the WB method. In contrast, higher SOM values than those were obtained by using the LOI method, irrespective of soil type or land use. On the one hand, this could mean that the content of oxidizable organic matter in the soils of these arid environments is low or very low comparing with the recalcitrant and/or stabilized organic compounds resistant to the sulfochromic attack of the WB method. This result may indicate a possible underestimation of SOM by WB as a result of the use of the Van Bemmelen factor for the calculation of SOM. It is known that recalcitrant organic matter has more carbon and less C:N ratio than the labile one (Dungait et al., 2012).

In fact, the environmental conditions have probably influenced and acted on the soil easily degradable organic matter and, mainly recalcitrant organic compounds can resist in the soil. As a consequence, poor labile organic matter is available to be used by soil biota and plants as a source of nutrients, resulting less fertility in these arid regions (Table 2). In the other hand, a high overestimation with the LOI methodology was probably obtained. It is generally accepted that some overestimation would be possible when the LOI is used as a method to estimate the organic content in soils and sediments, but the differences are too significant in the analysed soils to consider this as the only cause of the difference between both methods. The studies that have been carried out by other authors (Abella & Zimmer, 2007; Jensen et al., 2018; Vahel et al., 2017), considered that the LOI methodology overstate the organic matter content in soils, especially in those with a high clay content. According to El Mouridi et al. (2023), clay soils have the potential to retain more moisture and have structural water, which is then released in the form of water during the heating process of the soils. When the LOI method is used, which is based on weight difference, this moisture that remains after drying soils at room temperature even in desert areas, maybe still presented in the samples and may cause some overestimation of SOM. Regarding other interferences of LOI, the temperature used in our case was low to avoid the effect of loss of volatile salts, or metal oxides, or inorganic carbon. The presence of a low oxidizable organic matter content opposite to a more recalcitrant and stabilized fraction could be an additional effect determining the differences observed.

The results of our study indicated a significant variation between the values obtained through the LOI method and those derived from the WB titration method. However, the conversion of SOM estimated by LOI to SOC is not recommended, although the ratio LOI-SOM/TOC is considered about 2 (Bojko & Kabala, 2014) and can be used for TOC estimation. In fact, this value is higher than the factor 1.724 commonly used in WB method. Because of the facilities, LOI may be used in some labs as an easy technique to measure SOC using a conversion factor (Jensen et al., 2018), although there is not a commonly accepted factor to transform soil organic matter into organic carbon.

### Relationship between the two methods

**Fig. 3:**
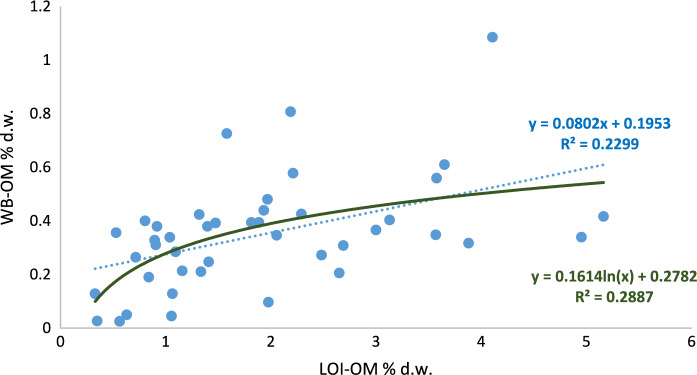
Square Pearson's correlation coefficient and relationship between the two organic matter estimation methods (WB-OM: Walkley–Black method; LOI-OM: loss on ignition) for all the samples and land uses land (blue dot line: linear adjustment, dark green continues line: logarithmic adjustment)

**Fig. 4:**
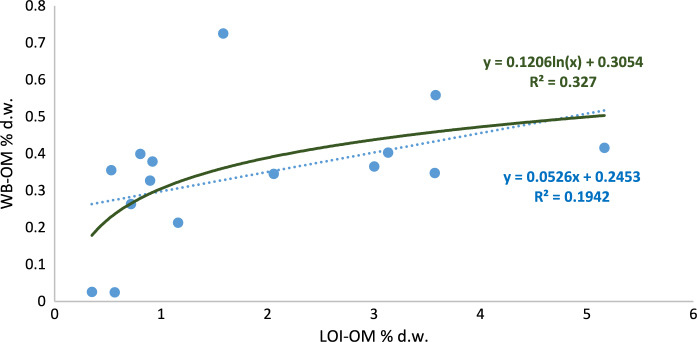
Square Pearson’s correlation coefficient and relationship between the two organic matter estimation methods (WB-OM: Walkley–Black method; LOI-OM: loss on ignition) for agricultural land (blue dot line: linear adjustment, dark green continues line: logarithmic adjustment)

**Fig. 5:**
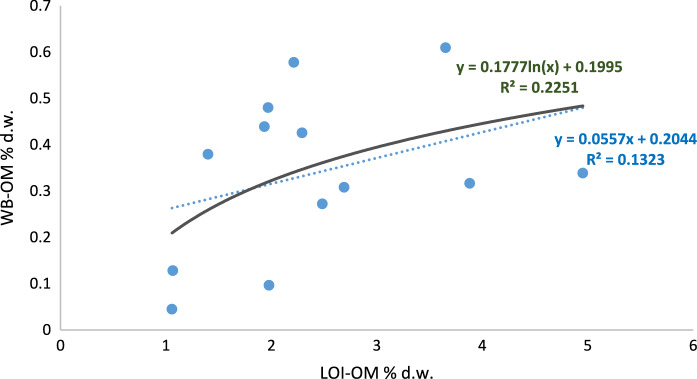
Square Pearson’s correlation coefficient and relationship between the two organic matter estimation methods (WB-OM: Walkley–Black method; LOI-OM: loss on ignition) for forest land (blue dot line: linear adjustment, dark green continues line: logarithmic adjustment)

Nevertheless, it would be expected a direct relation between both methods, considering a proportionality between oxidable organic matter and total organic matter (Gelman et al., 2012). For this reason, Square Pearson's correlation coefficient (R^2^) was employed to assess the correlation between the two methods across all samples and for each type of LULC (Figs. 3, 4, 5). But the results resulted in low values ​​of this coefficient, R^2^ = 0.23 (Fig. 3). When logarithmic correlation was tested, the results for all the samples led to a better correlation, but the coefficient was still low (0.29).

**Fig. 6:**
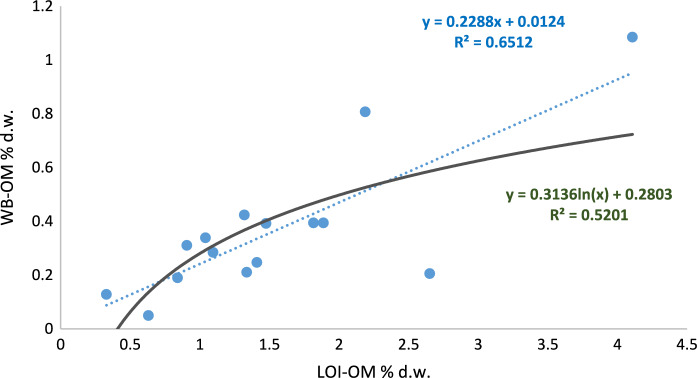
Square Pearson’s correlation coefficient and relationship between the two organic matter estimation methods (WB-OM: Walkley–Black method; LOI-OM: loss on ignition) for pastoral land land (blue dot line: linear adjustment, dark green continues line: logarithmic adjustment)

It can be observed that most of the soils reveal low or very low levels of organic matter content; 65% of the soils had less than 2% of SOM when using LOI and, 91% of them had less than 0.5% of SOM when WB method was used. The LOI-WB linear regression models revealed that for agricultural and forest land, a low correlation, R^2^ = 0.19* and 0.13* respectively, was found (Figs. 4, 5), while for pastoral land showed a better correlation (R^2^ = 0.65***) as it can be seen in Fig. 6. This would mean that the relation between both methods depends on land use and soil management. The amount of fresh organic matter added to the soil, as amendment, animal droppings or plant residues, would determine the evolution and accumulation of organic matter in the soil profile and, those factors differ for each type of land use. Oxidation-resistant organic carbon stored in the soil is a relatively stable organic matter (Mikutta & Kaiser, 2011) and the accumulation of SOM in the soils is favoured by the presence of this recalcitrant fraction of the organic matter (Navarro-Pedreño et al., 2021). This organic matter can resist the oxidant-acid attack done during the WB method but would be affected by LOI, during burning period, and can be estimated by this method, especially considering the prolonged time of 8 h burning used in this research.

As it was presented in the previous charts, the best correlation was obtained in the case of pastoral land use considering the linear regression (Fig. 6). However, when the logarithmic regression was applied (dark green line in the figures), both agricultural and forest land uses slightly increase the R^2^ but opposite of this, it was reduced in the case of pastoral land use. It is not easy to explain this behaviour, but probably when the SOM is over a determined value, close to 0.5% (in dry weight) in the case of WB, a linear regression is adequate and, an adjustment similar to that was found by Gelman et al. (2012). If we consider the differences between mean SOM values estimated by LOI and those determined by WB method (ΔSOM), these differences were higher in the forest soils (Table 3). Does this mean that the forest soils of this area have more stabilized organic matter than the soils affected by farming and grazing? In some way, these results could reinforce the idea that forest soils stored more stabilized organic matter than the other land uses even in arid environments, as occurs in temperate temperature zones and tropical regions.

**Table 3:** Mean values and standard deviation of the differences of soil organic matter (ΔSOM = LOI-WB)

	Agricultural	Forest	Pastoral
ΔSOM-mean value	1.52	2.09	1.17
St.dev	1.40	1.09	0.74

*St.dev* standard deviation

The different type of organic matter in the soil would be associated to the land use, considering that the soils are under the same environmental conditions in this case. Modifications in soil management, vegetation cover and land use are likely to increase or decrease SOM, and contribute to the accumulation or not of organic carbon in soils (Jones et al., 2005; Post & Kwon, 2000; Powlson et al., 2014; Schulze et al., 2009), where the accumulation was especially noticed following conservation strategies. However, many factors influenced the SOM. For instance, factors such as the physical protection of SOC against decomposition, which includes the spatial inaccessibility of organic matter to decomposing organisms and stabilisation by the interaction with mineral surfaces, play a crucial role in contributing to the spatial heterogeneity of SOC stability (Tian et al., 2016).

In this study, under arid conditions, the analysed sites subjected to different management practices and land use showed variations, and the total organic carbon stored in the soils decreased in the following order: Forests > Pastoral > Cropland when SOM is determined by using LOI. However, when WB methodology was used to determine SOC and after that, calculated the SOM applying the “Van Bemmelen factor”, the values between all of them are so close and poor differences were observed (Table 2). Moreover, Forest soils gave the less mean value with WB methodology. This suggested the idea of the presence of more stabilized and recalcitrant organic matter in the forest soils without less man disturbance as it happen in agricultural and pastoral soils, where the addition of fresh/non stabilized organic matter occurs because of the land management (amendments or animal droppings). The most recalcitrant organic matter, with a higher carbon content and therefore more carbonaceous, suffers less intensely from the acid and chromic oxidative attacks of the WB method, while it is possible that its combustion occurs using LOI.

In the other hand, high clay content facilitates the formation of humus-clay complex protecting, in some way, the organic matter to the acid and chromic attack of the WB method. The differences between both methods were very important and probably, the effect associated to clay minerals and remaining water content cannot explain these differences in this arid environment and these sandy soils. These results reinforce the idea of the need of having a standard procedure to check the SOM, easy and affordable for most of the soil researchers and farmers in arid environments. For instance, paying attention to the results given by LOI, those are very important and confirm that agriculture can decrease the SOM in arid regions comparing with forest soils as in temperate regions, and this means that it is necessary to improve the soil management practices to protect SOM and promote the conservation agriculture to increase SOC. However, we cannot assume this taking into consideration the WB results and this can lead to contradictory results. It is widely assumed that agriculture in arid Mediterranean environments facilitates the loss of soil organic matter, unless measures are taken to conserve and maintain organic matter levels. This fact is accentuated in the interior regions close to or included in the Sahara. Although most of the soils analysed showed low and very low values of soil organic matter, this is not the only parameter to be considered for knowing the good condition of soil regarding good and healthy soil management practices. Many properties are affected by the presence of organic matter, like soil erodibility which is of big concern regarding soil losses (Othmani et al., 2023). Nevertheless, it is necessary to ensure the implementation of soil conservation practices based on the storage of organic matter content in the soil profile as a general rule, this overarching approach can effectively contribute to minimizing soil losses and mitigating climate change.

It is very criticisable and should be discussed, because it can vary for different type of soils and environments. According to Abbas et al. (2020) findings, which confirm the need for specific agricultural practices within arid and semi-arid regions, characterized by high temperatures and low rainfall conditions, to effectively preserve and restore soil organic carbon while mitigating soil erosion, in contrast to the strategies employed in Mediterranean and tropical regions, the methods to estimate SOC/SOM should be adapted to these arid regions. The study of organic matter is essential for understanding how various activities influence soil carbon sequestration and contribute to global climate change (Liu et al., 2019). This understanding is crucial for effectively addressing global climate targets, and it is recommended that estimates global, regional and local carbon budgets should be obtained to quantify the net global warming potential. To ensure accuracy and reliability, these models must be calibrated to local conditions and always rely on direct measurements from physical soil samples, where SOC and bulk density have been directly measured.

## Conclusions

The work carried out in the Ghardaïa region (Algeria) enabled us to study the amount of organic carbon presented in the soils (0–30 cm depth) of this arid region under different types of land use, taking into account that the arid climate and the sandy soils are the common factor for all of them. The estimation of soil organic carbon (SOC) by using mass loss during ignition (LOI) is an available option for measuring SOM because of the simplicity of LOI and the possibilities of its application worldwide more than other modern techniques, including in developing countries.

The results demonstrated that the values obtained by the WB titration method, differed significantly from those obtained from LOI method. Even considering the overestimation of LOI, it seems that SOM could be better determined (recalcitrant/stabilized and oxidizable matter) by this method. It should be noted that the results and conclusions of the study are specific to the Saharian region of Ghardaïa and may be generalizable to other arid regions in Algeria or elsewhere, considering similar environmental conditions and soil types. The methods for determining soil organic matter should be adapted to the local conditions and in the case of WB, the conversion factor from oxidizable organic carbon to SOM would be revised for this type of environments and soils. Although many soil properties can affect the presence of organic matter and even the methodologies to check the SOM, it is of special interest when using LOI because it is necessary to control the temperature to avoid influence from soil inorganic carbon compounds and gypsum. However, LOI as a routine procedure, gives easy and quick results to compare soils and, moreover, without using pollutant chemical agents based on chromium as it occurs in WB. This is a highly environmental recommendation to reduce the presence of chromium species in the environment as chromium is a serious pollutant (Sharma et al., 2022). In the other hand, high clay content facilitates the formation of humus-clay complex protecting, in some way, the organic matter to the acid and chromic attack of the WB method.

The differences between both methods were very important and probably, the frequent overestimation with LOI cannot explain these differences in this arid environment and these sandy soils. Also, the recommendations given in the literature were followed in this work and the temperature used was low enough to avoid carbonates decomposition but optimum to burn organic carbon. The conventional dry combustion or wet oxidant-acid attack methods for SOM determination are the most widely used. Nevertheless, these results can serve as a basis for further studies and contribute to a better understanding of organic carbon measurements and organic matter stored in hot and arid regions of the world, where soils usually have low soil organic matter. A better understanding of spatial and temporal variations in management practices is essential for knowing the evolution of soil carbon stocks on a global scale, including desert regions where poor data is available, and strengthening our ability to develop effective adaptation strategies for the mitigation to climate change.

## Data Availability

Not applicable.
